# Design, Fabrication, and Evaluation of 3D Biopotential Electrodes and Intelligent Garment System for Sports Monitoring

**DOI:** 10.3390/s24134114

**Published:** 2024-06-25

**Authors:** Deyao Shen, Jianping Wang, Vladan Koncar, Krittika Goyal, Xuyuan Tao

**Affiliations:** 1 College of Fashion and Design, Donghua University, Shanghai 200051, China; deyao.shen@ensait.fr; 2 École Nationale Supérieure des Arts et Industries Textiles—ENSAIT, ULR 2461—GEMTEX—Génie et Matériaux Textiles, University of Lille, F-59000 Lille, France; vladan.koncar@ensait.fr; 3 Key Laboratory of Clothing Design and Technology, Donghua University, Ministry of Education, Shanghai 200051, China; 4 Shanghai Belt and Road Joint Laboratory of Textile Intelligent Manufacturing, Shanghai 200051, China; 5 Department of Manufacturing and Mechanical Engineering Technology, Rochester Institute of Technology, Rochester, NY 14623, USA; krgmet@rit.edu

**Keywords:** 3D biopotential electrode, intelligent garment system, sports monitoring, physiological signal

## Abstract

This study presents the development and evaluation of an innovative intelligent garment system, incorporating 3D knitted silver biopotential electrodes, designed for long-term sports monitoring. By integrating advanced textile engineering with wearable monitoring technologies, we introduce a novel approach to real-time physiological signal acquisition, focusing on enhancing athletic performance analysis and fatigue detection. Utilizing low-resistance silver fibers, our electrodes demonstrate significantly reduced skin-to-electrode impedance, facilitating improved signal quality and reliability, especially during physical activities. The garment system, embedded with these electrodes, offers a non-invasive, comfortable solution for continuous ECG and EMG monitoring, addressing the limitations of traditional Ag/AgCl electrodes, such as skin irritation and signal degradation over time. Through various experimentation, including impedance measurements and biosignal acquisition during cycling activities, we validate the system’s effectiveness in capturing high-quality physiological data. Our findings illustrate the electrodes’ superior performance in both dry and wet conditions. This study not only advances the field of intelligent garments and biopotential monitoring, but also provides valuable insights for the application of intelligent sports wearables in the future.

## 1. Introduction

In recent years, the rapid advancement of wearable technology has opened a new era for continuous health monitoring and bioelectrical signal analysis [1]. Wearable devices have become important in providing real-time bioinformation, enhancing communication, and enabling health monitoring [2,3]. This evolution has been made possible through the integration of sophisticated textiles, materials, and microelectronics [4].

A critical aspect of wearable technology involves the monitoring of electrophysiological signals such as ECG, EMG, etc., to assess human physiological conditions [5,6,7]. Traditional clinical long-time monitoring practices have relied heavily on disposable adhesive silver/silver chloride (Ag/AgCl) electrodes for capturing these signals [8]. However, the practicality of these electrodes for long-term, continuous monitoring presents significant challenges. Despite the remarkable progress in wearable technology, the limitations of existing electrode technology remain a significant concern. The use of Ag/AgCl gel electrodes, while effective in the short term, is hindered by their inherent drawbacks. Skin irritation, caused by prolonged contact with the gel, poses discomfort and can lead to allergic reactions [9]. Moreover, the gel’s tendency to dehydrate over time results in the degradation of signal quality, rendering it unsuitable for long-term or daily use [10]. To address these issues, researchers have diligently sought alternatives, leading to the development of dry electrodes using conductive elastomeric materials [11,12,13]. Among these, conductive elastomeric materials have gained prominence, alongside innovative compositions such as Ag/G (silver-coated glass) composite materials [14], PDMS-CB (Polydimethylsiloxane-Carbon Black) conductive polymers [15,16], and PEDOT:PSS (poly(3,4-ethylenedioxythiophene) polystyrene sulfonate) conductive composites [17,18,19]. The design of semi-dry electrodes represents a significant enhancement to these developments, combining the hydration control of wet electrodes with the user-friendly attributes of dry types. These semi-dry electrodes utilize superporous hydrogels to regulate electrolyte release through capillary action, effectively maintaining stable skin-to-electrode impedance and enhancing user comfort for extended wear [20,21]. By incorporating materials like polyacrylamide and polyvinyl alcohol, these electrodes effectively reduce impedance and improve signal fidelity [22,23]. This evolution illustrates a significant shift towards creating more adaptable electrodes that not only offer reduced skin irritation and improve wearer comfort, but also maintain high conductivity and signal fidelity for ECG/EMG/EEG monitoring, offering substantial advantages over traditional Ag/AgCl electrodes [24]. In comparison to traditional Ag/AgCl electrodes, dry electrodes offer significant advantages in terms of their ease of use and non-invasiveness for electrocardiogram (ECG) monitoring. These electrodes, characterized by their lack of requirement for gel or other wetting agents, enable a quicker setup and are conducive to repeated long-term use without the risk of skin irritation common with their Ag/AgCl counterparts. However, despite their promise for revolutionizing ECG monitoring, dry electrodes encounter distinct challenges. Among these, the issues of breathability and comfort during extended periods of sporting activities are particularly notable. The integration of these electrodes into wearable technology for continuous health monitoring necessitates innovative solutions to enhance their adaptability and user experience, especially in dynamic and physically demanding environments.

In recent years, the domain of textile electrodes has witnessed a notable surge in scholarly interest, particularly regarding electrodes fabricated from silver threads. This heightened focus is largely due to the distinctive attributes of textile materials, which include inherent softness and comfort, coupled with their facile integration into wearable devices. Silver yarn electrodes, characterized by their superior electrical conductivity, stand out as a promising avenue for advancement in wearable technologies. As for the realization of a conductive flexible surface for electrodes, such as compression garments, the most used technologies are screen-printing [25,26,27], coating [28,29], and embroidery [30,31]. However, they demonstrate some significant limitations. For instance, the durability of printed electronics on fabrics is a significant concern, particularly their ability to withstand the rigors of bending, stretching, abrasion, and repeated washing cycles [32]. Furthermore, dip-coating procedures exhibit inconsistencies in the surface texture and uniformity of conductive layers applied to textiles, which can result in fluctuations in electrical resistance. Control over the thickness of these layers is inherently limited, as it is contingent on the textile’s surface morphology, tension within the substrate, and a range of processing parameters—time, temperature, withdrawal speed, compound concentration, and the composition of the coating bath [33]. Embroidery technology, while adept at creating dense conductive surfaces, tends to produce structures that are excessively thick and rigid, which are consequently lacking in essential flexibility and stretchability [34].

In contrast, electrodes developed through knitting technology not only preserve the fabric’s natural elasticity and comfort but also seamlessly integrate electronic functionalities, ensuring minimal impact on the textile’s inherent stretchability and softness. This method proves especially advantageous for compression garments, where maintaining the original textile properties while incorporating electronic capabilities is crucial. Furthermore, the advancement in 3D knitted electrodes structure introduces a significant innovation in smart textile fabrication. This structure is inherently designed to accommodate complex shapes and functionalities, enabling a direct integration of electronic features into the textile’s architecture and offering a one-step process that enhances the efficiency and feasibility of producing smart textiles. This methodology not only streamlines the manufacturing process but also opens new avenues for creating more sophisticated and integrated wearable technologies.

**Figure 1:**
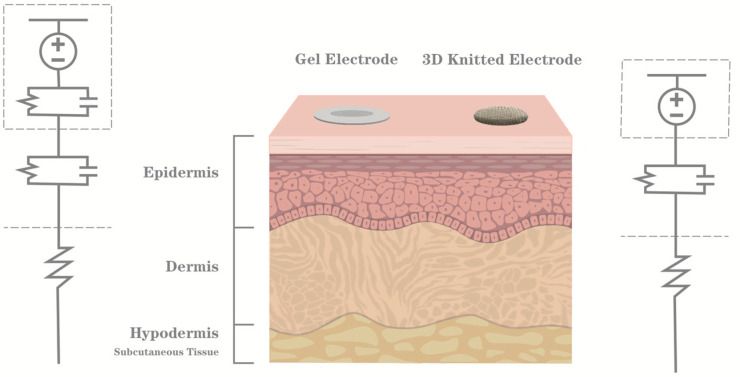
Skin contact model for gel and dry electrodes.

Our research focuses on the innovative design and fabrication of 3D knitted silver electrodes, tailored for real-time biopotential signals monitoring during long-term physical activities. As shown in Figure 1, compared to gel electrodes (left side), dry electrodes (right side) are more concise in the skin contact model, and due to the abandonment of gel, sweat can be a better conductive medium, and the conductivity of dry electrodes does not decrease as much as that of gel electrodes along the increase of the exercise duration. On the left side of Figure 1, two RC parallel cells can be observed: one of them illustrates the skin-to-electrode impedance and the other indicates the gel impedance. When the gel dehydrates, the corresponding impedance in the RC cell increases strongly, making it very difficult for the biosignal acquisition. On the right side of Figure 1, there is only one RC cell for the skin-to-textile dry electrode impedance. Furthermore, because of the 3D structure, the physical contact between the electrode and the skin is improved compared with traditional coating/embroidered textile electrodes. Therefore, 3D knitted silver electrodes can help us to enable extended, real-time physiological signal monitoring while ensuring user comfort and ease of use. To achieve this goal, we have leveraged advanced silver fiber materials, a leading-edge solution in the field. Through a systematic exploration of various electrode parameters, such as height, size, and pressure, we have conducted skin-to-electrode impedance experiments to determine the optimal electrode design. By addressing the existing limitations of dry electrodes, our work paves the way for practical, real-time, and continuous physiological monitoring. This development holds considerable promise for applications in healthcare, sports, and everyday life, with potential implications for the early diagnosis and management of various conditions.

This study aims to develop an intelligent garment system with 3D knitted electrodes to collect the ECG and EMG signals for long-term monitoring. Our 3D electrodes guarantee the low skin-to-electrode impedance and therefore good-quality biosignals acquisition. Arduino Nano 33 IoT-based hardware has been designed and employed to collect and send the data to a remote database. The structure of our study unfolds over several sections to intricately detail the creation and evaluation of an intelligent garment system for sports monitoring. Section 2 elaborates on the design process, including the fabrication of 3D biopotential electrodes, the construction of the intelligent garment, and the specifics of the hardware design for data acquisition and transmission. Section 3 examines the method and outcomes of impedance measurement, validating the low skin-to-electrode impedance facilitated by our 3D electrodes for superior biosignal quality. In Section 4, we present biomeasurement results from cycling activities, demonstrating the system’s capability in capturing precise ECG and EMG signals. The discussion in Section 5 reflects on these findings, considering their implications for the advancement of sports monitoring technologies. This article concludes in Section 6 by summarizing the contributions of our study to the development of intelligent garments for enhanced sports performance analysis.

## 2. Materials and Structures

### 2.1. Three-Dimensional Biopotential Electrodes Fabrication

#### 2.1.1. Materials

**Figure 2:**
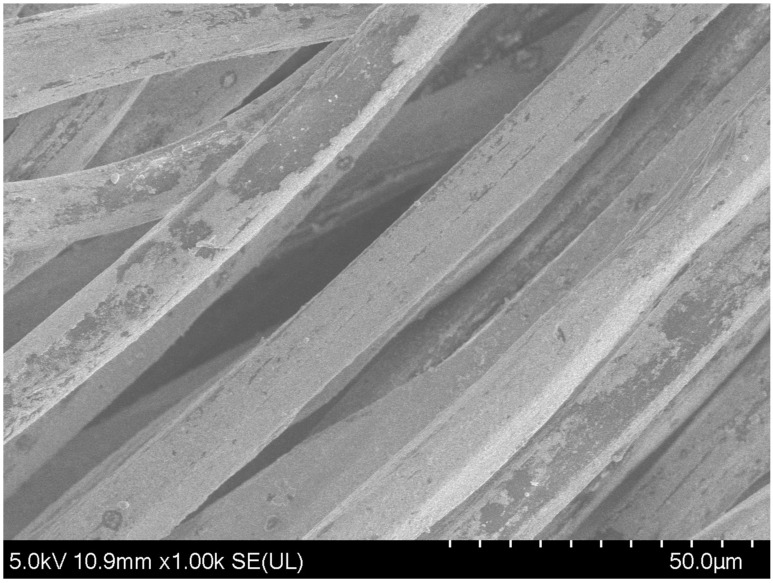
SEM of low silver-plated nylon thread.

In this paper, 3D knitted silver electrodes are knitted by using silver-plated nylon thread (DTY 140D/3 Silver Fiber Filament), which is provided by Kazhtex Technology Co., Ltd. (Suzhou, China). Figure 2 shows the SEM (scanning electron microscope) images of the silver-plated nylon thread, showcasing its microstructure. The thread features a diameter of 0.45 mm and a resistance of 200 Ω/m. Additionally, it is composed of three-ply yarn integrating 48 filaments, utilized for knitting the 3D electrodes. For the other components of the intelligent garment system except electrodes, a high-elasticity nylon filament, made up of a blend of 20D spandex and 40D nylon with a spandex stretch ratio of 3.6, is employed. This filament is supplied by Yinrui Fiber Company (Shaoxing, China) and is specifically applied for knitting the plain stitch structures of the garment.

Additionally, regarding the connection between the 3D knitted silver electrodes and ADS1292R integrated circuit and EMG sensors, we have opted for customized composite silver conductive yarns. The core yarn of this conductive yarn is composed of 18 strands of silver-plated nylon filament with a high silver content, exhibiting an electrical resistance of less than 2.7 Ω/m and exceptional conductivity. An insulating layer encases the core to prevent conductivity issues with other wires, while the outermost layer consists of a braided silver layer serving as a shield. This design aims to minimize the influence of power frequency interference on the collected ECG/EMG signals, thereby ensuring the acquisition of clearer and more stable signals.

#### 2.1.2. 3D Biopotential Electrodes Fabrication Structure

**Figure 3:**
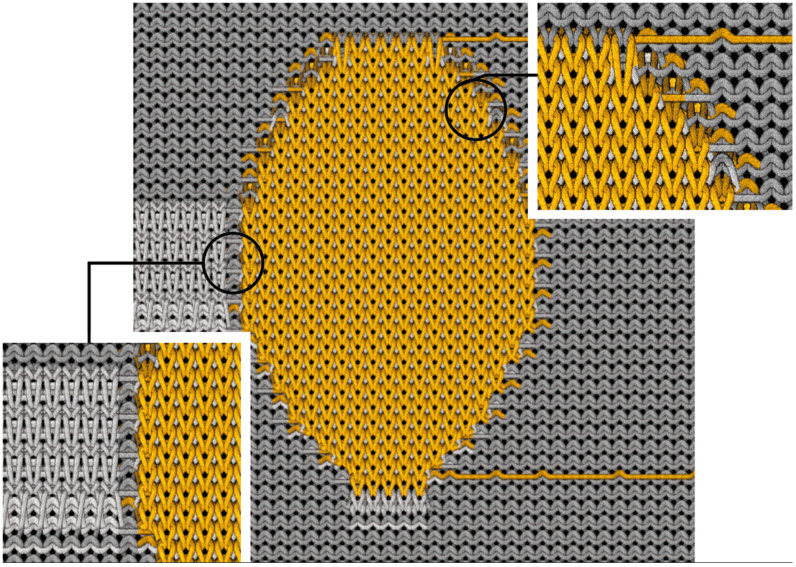
Structure of 3D knitted electrodes on the back side of fabric.

The textile electrodes are fabricated on a STOLL 72G knitting machine (STOLL, Reutlingen, Germany). The base knitted fabric is jersey stitch using a high-elasticity nylon filament. This choice of stitch is predominantly favored for manufacturing compression garments. Figure 3 illustrates the structure of the 3D knitted silver electrode, which necessitates a harmonious combination of diverse knitting textures to achieve the envisioned complex 3D construct. The electrode component, marked by the yellow color, employs silver-plated nylon thread and is meticulously knitted as a round shape using the double tuck stitch. This specific knitting technique enhances the conductivity of the electrodes, enabling a localized 3D effect.

**Figure 4:**
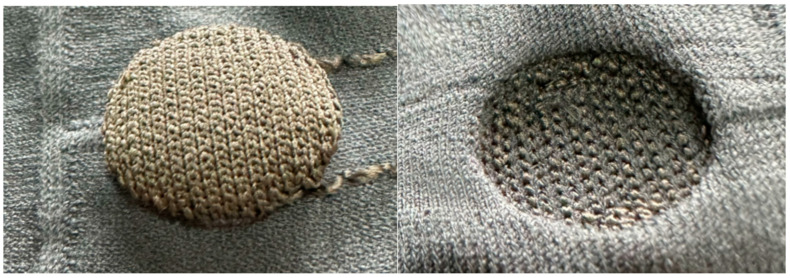
Three-dimensional knitted silver electrode. (**Left**) Back side of the knitted fabric. (**Right**) Front side of the knitted fabric.

The silver-plated nylon thread is observable, floating on the back surface. It is introduced and exited (“up in, down out”) during the knitting of the 3D electrodes, floating above the back surface of the jersey knit structure. This floating is called drop stitch structure, where the threads do not integrate into the primary knitting structure. To prevent excessive length leading to potential thread breakage or needle collision in the machine, double tuck stitches are employed at intervals to ensure the knitting process proceeds smoothly. In the magnified view at the top right corner of Figure 3, the transition between the two types of yarn is depicted, showcasing the application of localized intarsia techniques to intertwine the differing yarns seamlessly. The high elasticity of the nylon filament, contrasted with the non-elastic nature of the silver-plated nylon thread, along with the difference in knitting densities, collaboratively produce a significant 3D effect. Additionally, by adjusting the yarn tension in the STOLL flat knitting process, we are able to govern the height of the electrode. Increased yarn tension results in a more pronounced electrode protrusion, thereby amplifying the 3D effect and enabling precise control of the electrode’s 3D attributes. This localized intarsia approach also aids in enclosing the edges of the electrodes, ensuring the stability of loop dimensions and preventing unraveling due to stretching or other external forces. Additionally, a magnified view of a knitted hollow channel located in the lower-left corner of Figure 3 demonstrates the creation of this channel through drop stitch techniques. This channel is specifically engineered to house low-resistance silver conductive wires, which function as a medium for connecting the 3D silver electrodes to electronic components. The 3D knitted silver electrodes are displayed in Figure 4.

**Figure 5:**
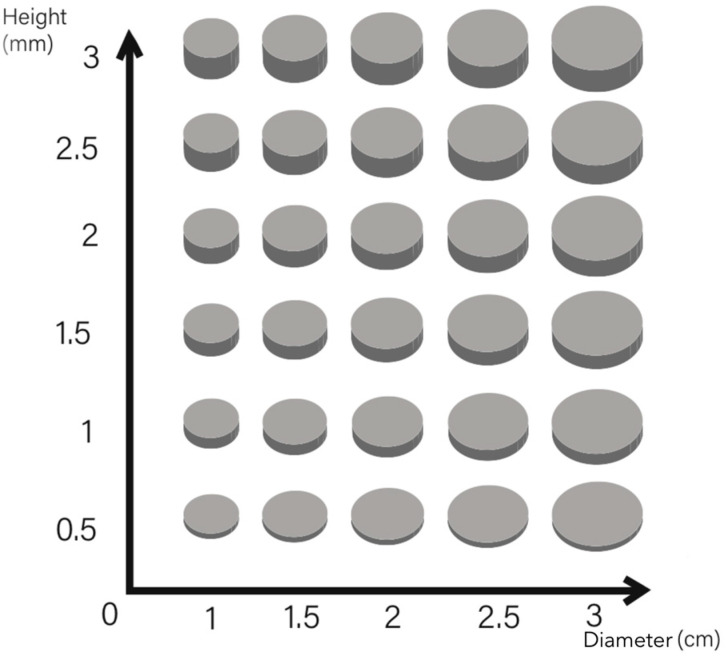
Parametric design of 3D knitted electrodes by height and diameter.

Figure 5 presents the variations in electrode diameters and heights explored for impedance measurement. The diameter of the electrodes range from a minimum of 1 cm to a maximum of 3 cm, with increments of 0.5 cm, a range chosen for its suitability in bioelectrical signal detection. The height of the electrodes varies from 0.5 mm to 3 mm, also in increments of 0.5 mm.

### 2.2. Garment Design

**Figure 6:**
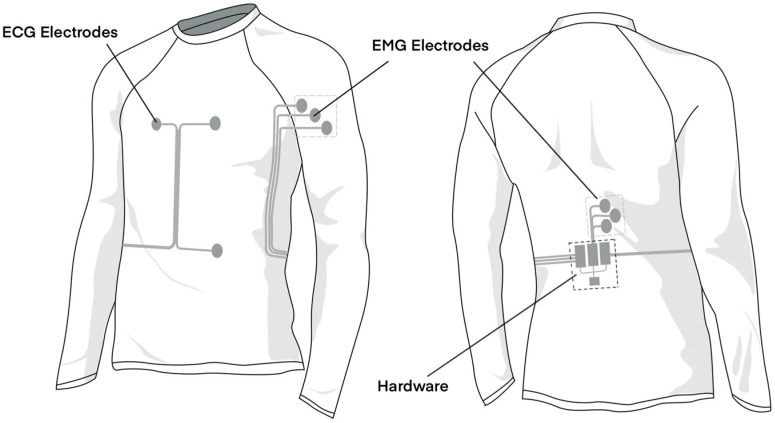
Design of intelligent garment system.

The design of the intelligent garment is depicted in Figure 6. This garment incorporates two electrodes at the chest area and one at the abdomen for monitoring ECG signals through a single-lead approach. Recognizing that the deltoid muscle on the arm and the erector spinae on the back are muscles prone to fatigue during sports, we strategically placed three electrodes at each of these locations to monitor EMG signals. Following the guidelines of the Consensus for Experimental Design in Electromyography (CEDE) project, the choice of electrode type for these muscles was meticulously considered [35]. For the deltoid anterior and erector spinae, we chose 3D knitted silver electrodes. These electrodes are selected for their innovative integration with the fabric, which maintains a consistent connection with the skin despite the dynamic and complex movements during sports, thereby ensuring the reliability and accuracy of EMG signal measurement without compromising the athlete’s comfort. Additionally, sensors and Arduino Nano 33 IoT are positioned on the lower part of the back, a location determined to be the most stable during sports activities, thus minimizing displacement caused by motion and reducing the impact on the wearer’s bodily perception, thereby not compromising comfort during riding. For the connection between sensors and electrodes, we employed the previously mentioned channel design, wherein low-resistance silver conductive wires were placed within the channels to achieve a flexible connection. This design allows the wearer to barely feel the presence of the wires, representing an innovative attempt to balance comfort with functionality.

**Figure 7:**
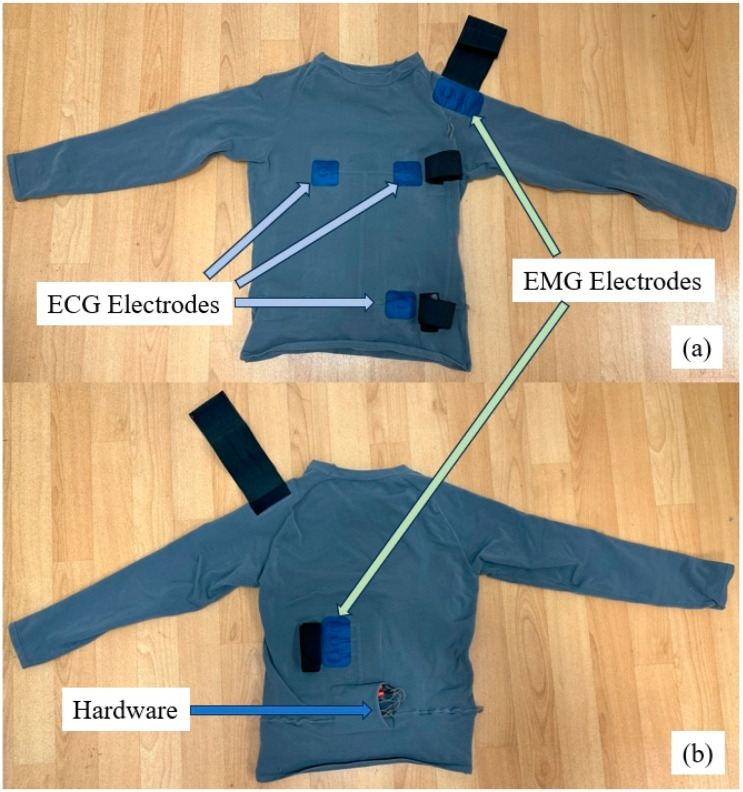
Front and back views of intelligent garment system. (**a**) Front side of the garment. (**b**) Back side of the garment.

The intelligent garment system’s front and back views, as shown in Figure 7, incorporate the custom silver conductive wires we previously discussed for establishing connections, as well as channels designed to house these wires. As we can see, this innovative assembly is adeptly integrated into the system’s design. Additionally, the 3D effect of the electrodes creates a concave surface on one side, which under skin pressure can produce a convex bulge, detracting from the contact quality. To mitigate this, we utilized laser-cut sponges matching the electrodes in size and thickness, affixed with an insulating adhesive layer, ensuring dimensional stability and maintaining a close skin-to-electrode fit during use. According to the CEDE project’s recommendations, ensuring a stable and secure skin-to-electrode interface is crucial for maintaining signal quality, especially under dynamic conditions such as sports. Furthermore, adjustable Velcro straps have been strategically placed around the perimeter of each electrode on the intelligent garment system to regulate pressure. This adjustable feature follows the CEDE project’s advice on optimizing electrode performance through customizable fit, enhancing the overall functionality of the garment in recording accurate EMG signals. This design allows for enhanced signal acquisition, as a tighter fit results in improved electrode performance.

### 2.3. Hardware Design

**Figure 8:**
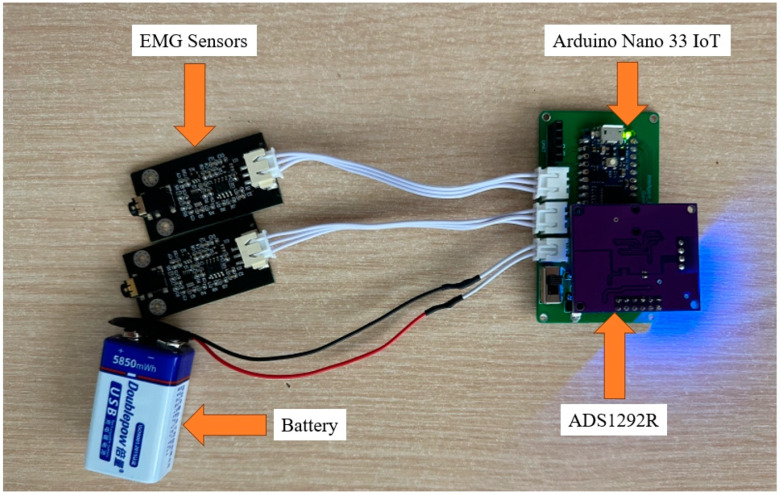
Hardware for intelligent garment system.

In terms of hardware, as illustrated in Figure 8, we utilized an ADS1292R chip for ECG monitoring, along with two sensors by OYMotion for EMG signal detection, which were interfaced with a development board equipped with an Arduino Nano 33 IoT, which was further supplemented by a 9 V battery to provide power. Arduino Nano 33 IoT features Wi-Fi connectivity, enabling the remote acquisition of real-time ECG and EMG data. This setup, incorporating Arduino Nano 33 IoT, enhances the seamless transmission of physiological signals, ensuring efficient and accurate monitoring within the intelligent garment system.

## 3. Impedance Measurement

### 3.1. Method

**Figure 9:**
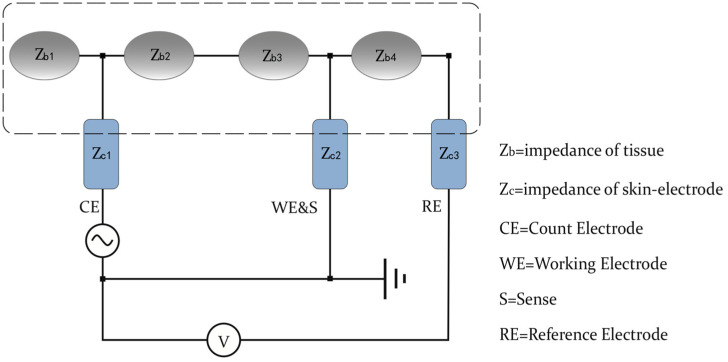
Circuit for impedance test.

The circuit diagram (Figure 9) is directly tailored to measure the impedance Z_c2_ of the skin-to-electrode contact in three-lead configuration. This inspiration came from the methodology presented in the article by Emanuel Gunnarsson [36]. The segment Z_b4_ is effectively isolated from carrying any current, as it is in series with the high impedance of the voltmeter. Consequently, the high-end potential of the voltmeter aligns with the junction where Z_b3_, Z_b4_, and Z_c2_ converge, specifically at one side of the skin-to-electrode contact Z_c2_, leading to the measurement equation V = Z_c2_ I. This methodological approach for impedance measurement does not depend on any presuppositions about the symmetry or precise knowledge of the tissue impedance, as these factors are extraneous to the calculation. The only prerequisite is that the voltmeter’s input impedance must be sufficiently high to prevent current leakage into the branch, a condition met by contemporary voltmeters. Thus, this configuration is uniquely suited for directly measuring the skin-to-electrode impedance without resorting to mere estimations, distinguishing it from other measurement setups. Moreover, this three-lead method overcomes the limitations of traditional two-lead approaches by not assuming uniformity of body and skin-to-electrode impedances across different anatomical areas. Focusing solely on the skin-to-electrode interface, it provides a direct and accurate measurement essential for the effective design and evaluation of textile electrodes in wearable technologies. This method ensures precise characterization of the skin-to-electrode interface, eliminating the need for assumptions about tissue symmetry or comprehensive tissue impedance knowledge, making it highly suitable for practical applications.

**Figure 10:**
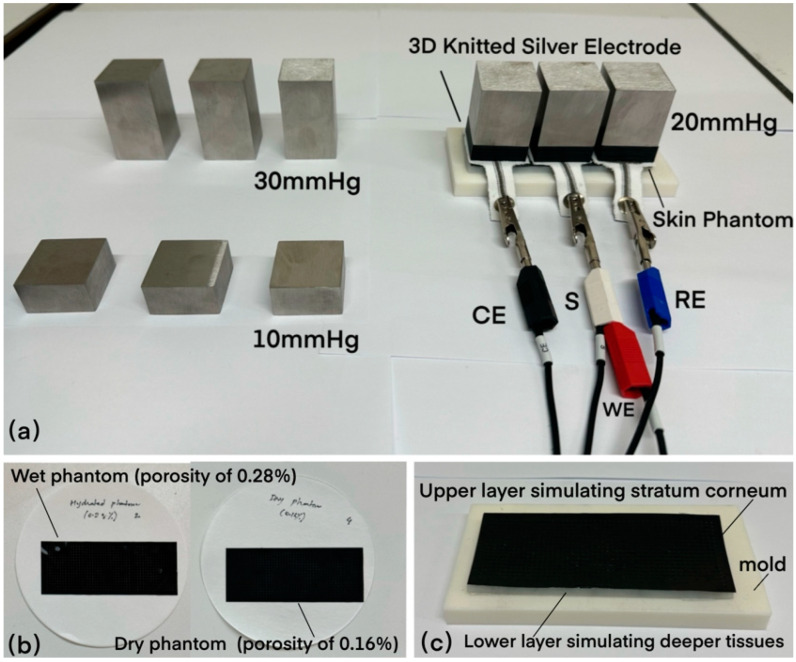
(**a**) Electrode–skin impedance test setup. (**b**) Wet phantom and dry phantom. (**c**) Upper and lower layers of the skin phantom and mold.

The Ivium-n-stat (Ivium Technologies Inc., Eindhoven, Netherlands) was employed for impedance measurements. The electrode–skin impedance test setup is shown in Figure 10a. To simulate skin pressure, we produced stainless steel blocks with pressure settings of 30 mmHg, 20 mmHg, and 10 mmHg [37,38]. These pressures are reflective of those used in medical-grade compression socks and provide a significant point of reference for evaluating compression garments. Skin phantoms made from agar have been widely used in the literature to evaluate the performance of biopotential electrode designs [39,40,41]. However, the existing phantoms have only been shown to be suitable for simulating the electrical characteristics of deeper tissue layers, such as the dermis and hypodermis, in a higher frequency range. Moreover, the skin hydration state cannot be modeled with the existing agar-based phantoms. Therefore, to model skin behavior during physical activity, a phantom mimicking the electrical properties of the skin was adapted from work by Goyal et al. [42], which can simulate the impedance of the skin in the frequency range 1 Hz–1000 Hz, which is significant for biopotential signal acquisition. In addition, it models skin hydration in a controlled manner by varying the porosity of the upper layer. The distinct porosities representing different hydration levels are evident in the phantoms depicted in Figure 10b, with the wet phantom (porosity of 0.28%) and the dry phantom (porosity of 0.16%) demonstrating the variability in simulating wet and dry skin conditions.

The skin phantom comprises two layers, as illustrated in Figure 10c: the upper layer, which represents the stratum corneum (the outer layer of the skin), and the lower layer, which represents the deeper tissue layers. The upper layer was fabricated by spin-coating a mixture of polydimethylsiloxane, 2.5% *w*/*w* carbon black (to increase conductance), and 40% *w*/*w* barium titanate (to improve dielectric properties) to achieve a 100 µm thick layer, emulating the electrical properties of the stratum corneum. For modeling skin hydration in a controlled manner, the porosity of the upper layer was varied, with 0.16% mimicking dry skin and 0.28% simulating wet skin impedance. The lower layer of the phantom representing the deeper tissue layers was fabricated using polyvinyl alcohol (PVA) cryogel solution and a freeze–thaw technique. The mixture was prepared using 8.8 g of PVA mixed with 0.9% *w*/*w* saline solution and a 5 mm thick layer was obtained.

### 3.2. Impedance Results

**Figure 11:**
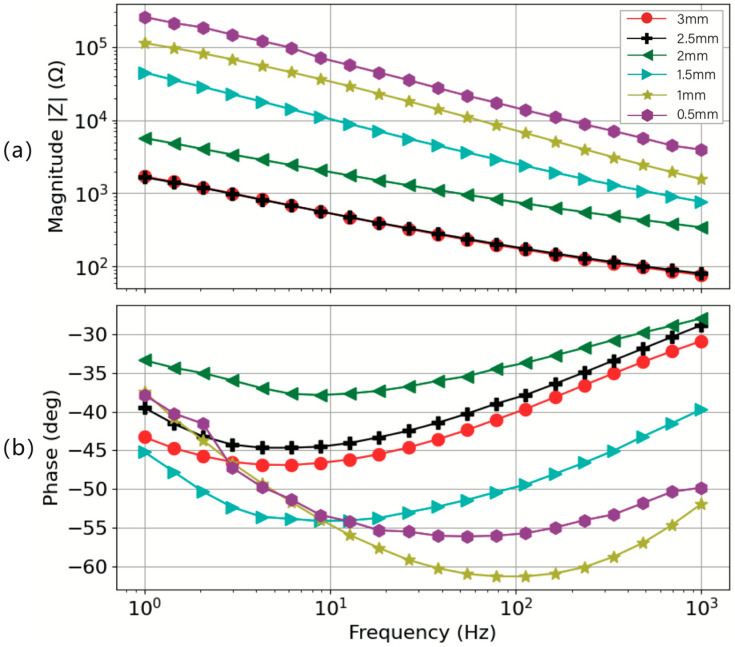
Impedance measurement result for the circle electrode with 3 cm as diameter and 0.5 to 3 mm as height under the pressure of 30 mmHg. (**a**) Impedance magnitude and (**b**) phase response.

Figure 11 shows the impedance–frequency characterization and phase–frequency characterization, performed under a constant pressure of 30 mmHg on a skin model with 0.28% porosity. It demonstrates two discernible trends: (1) for the same electrodes, its impedance decreases with increasing frequency; (2) for the same measurement frequency, the impedance of the electrode decreases with increasing height. This figure exhibits the impact of electrode height on impedance. These trends underscore the sensitivity of impedance to electrode geometry, which is critical for the design of devices intended for biopotential monitoring. Building on these observations, it becomes apparent that electrodes with smaller heights exhibit higher magnitudes of impedance. One potential reason for this trend may be that electrodes of greater height contain a higher quantity of silver-plated fibers; the contact area between the yarns inside the electrode is larger, which results in a denser internal structure under the same pressure, offering improved impedance. Consequently, this leads to lower impedance. Notably, electrodes with heights of 3 mm and 2.5 mm display remarkably similar impedance behaviors, consistently presenting lower impedance values across the frequency range from 1000 Hz to 1 Hz. The phase response for all the electrode configurations depicted a combination of resistive and capacitive behavior in the 1 Hz–1000 Hz frequency range, which is similar to the performance of dry electrodes on actual human skin. Moreover, the phase response for 3 mm and 2.5 mm height electrodes was not significantly different and was within 4 degrees of one another. This similarity indicates a potential threshold effect in impedance variation relative to electrode height, suggesting a diminishing return on impedance reduction beyond a certain electrode height.

**Figure 12:**
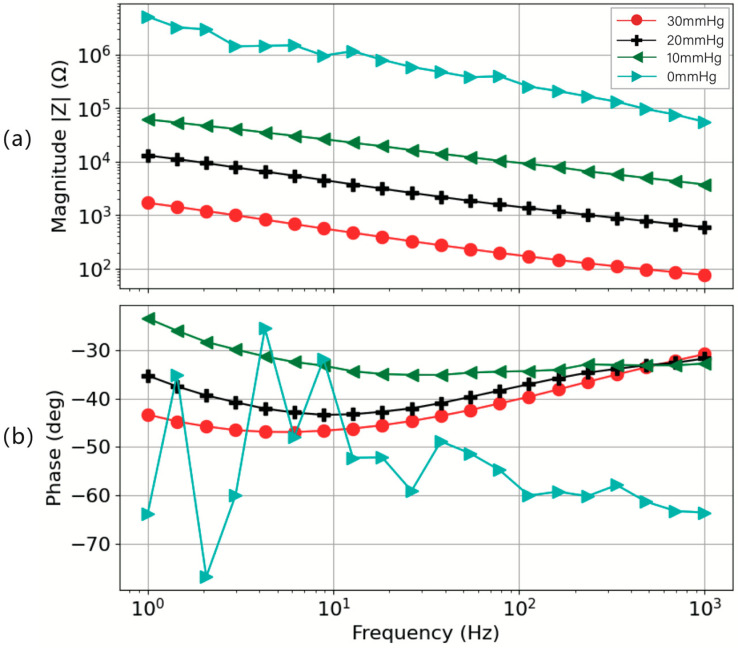
Impedance–frequency characterization and phase–frequency characterization, performed under controlled conditions featuring a 3 cm diameter circular electrode with a height of 3 mm and a skin model at 0.28% porosity. (**a**) Impedance magnitude and (**b**) phase response.

After understanding the trend of electrode height’s impact on impedance, we selected electrodes with a height of 3 mm for further exploration. The impedance magnitude and phase response of a 3 cm diameter circular electrode with a height of 3 mm applied to a skin model with 0.28% porosity display a clear increase in impedance with the reduction of applied pressure, as shown in Figure 12. The data illustrate a distinct variation in impedance under varying levels of applied pressure (Figure 12a). As the pressure exerted on the electrode decreases from 30 mmHg to 0 mmHg, the impedance magnitude correspondingly increases, indicating a pronounced pressure dependency. The color-coded plot effectively highlights this relationship, with the impedance incrementally rising as the pressure diminishes, a phenomenon that is especially marked at lower frequencies. The relatively higher skin-to-electrode impedance obtained (of the order of Mega ohms) for 0 mmHg pressure indicates that the contact was not sufficient between the electrode and the skin model. Further, the phase response for 0 mmHg indicates the dominant capacitive behavior (Figure 12b) compared to other phase responses obtained for higher pressures. This trend aligns with the findings of some scholars’ research [43,44]. This trend demonstrates the significance of contact pressure in biopotential signal monitoring for sports applications, where consistent and accurate signal capture is essential. The phase response obtained for 10 mmHg–30 mmHg of pressure was not significantly different for a 100 Hz–1000 Hz frequency range and was within 15 degrees for a 1 Hz–100 Hz frequency range. This indicates that with the increase in pressure, the effective skin-to-electrode contact was improved, thus resulting in lower skin-to-electrode impedance.

**Figure 13:**
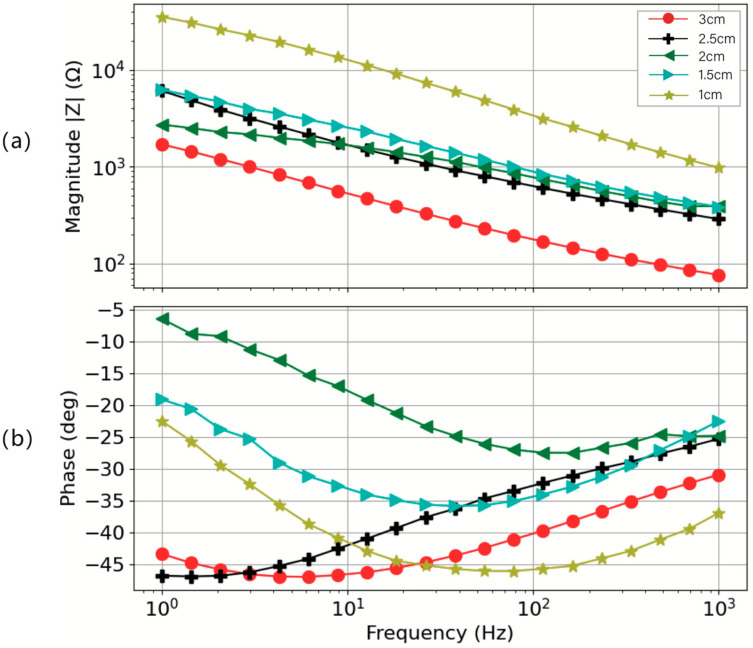
Impedance–frequency characterization and phase–frequency characterization, performed under controlled conditions featuring a height of 3 mm and a skin model at 0.28% porosity under the pressure of 30 mmHg. (**a**) Impedance magnitude and (**b**) phase response.

Figure 13 shows the impedance measurement results for a circular electrode with a height of 3 mm under an applied pressure of 30 mmHg. Figure 13a depicts the impedance magnitude, while Figure 13b illustrates the phase response associated with the electrode. Notably, as the diameter of the electrode increases, there is a corresponding decrease in impedance magnitude. One possible explanation for this trend is that electrodes with larger diameters have a greater surface area in contact with the skin and contain a higher silver content, thereby offering better electrical conductivity and resulting in lower impedance. This is in complete agreement with the previously reported work, as a higher-area electrode leads to lower impedance [45]. It is particularly worth mentioning that between diameters of 1.5 cm and 2.5 cm, the impedance variation was minimal. The phase response of 2.5 cm and 3 cm diameter electrodes was not significantly different and was within 5 degrees of one another. This trend of impedance homogeneity is most evident at frequencies below 10 Hz, where the differences in impedance are not pronounced, indicating a zone of diameter-related impedance stability.

**Figure 14:**
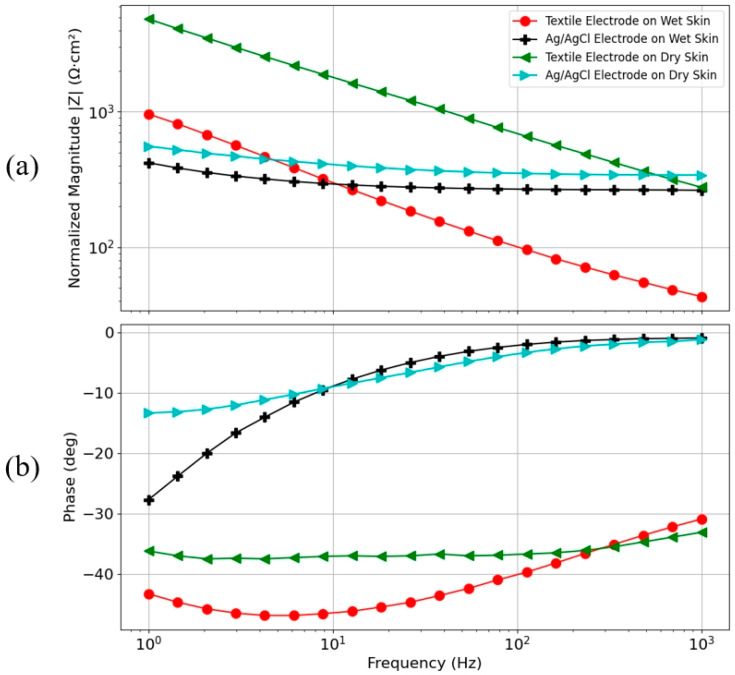
Impedance measurement results of 3D knitted silver electrodes and Ag/AgCl electrodes under dry and wet skin conditions. (**a**) Impedance magnitude and (**b**) phase response.

Figure 14 illustrates the impedance testing results of optimally parameterized 3D knitted silver electrodes compared to Ag/AgCl electrodes under both dry and wet skin conditions. To remove the influence of electrode area, the impedance values were normalized by the electrode area, termed normalized impedance, which provides a comparative measure independent of electrode dimensions [46]. The findings in Figure 14a reveal that, in tests with wet skin, the impedance of 3D knitted silver electrodes remains lower than that of Ag/AgCl electrodes across the frequency range of 10 Hz to 1000 Hz. However, at frequencies below 10 Hz, the impedance of the 3D knitted silver electrodes is higher. This difference can be attributed to the mechanistic principles of the electrodes; for Ag/AgCl electrodes, resistive coupling is dominant due to the charge transfer phenomenon facilitated by the presence of ionic gel. In contrast, dry electrodes exhibit either capacitive coupling or a combination of resistive and capacitive coupling between the electrode and skin in the low-frequency range, contributing to their higher impedance at frequencies below 10 Hz. During dry skin testing, Ag/AgCl electrodes exhibit higher impedance than 3D knitted silver electrodes near the 1000 Hz frequency, but at other frequencies, their impedance is lower. It is observed that the impedance curve of the 3D knitted silver electrodes has a steeper slope, indicating a significant increase in impedance as the frequency decreases from high to low. In comparison, the impedance of Ag/AgCl electrodes remains relatively stable, showing minimal frequency dependence, which is consistent with the dominant resistive behavior due to their charge transfer mechanism.

The phase response, depicted in Figure 14b, further illustrates the impedance’s frequency dependence by representing the combination of resistive and capacitive behavior between 1 Hz and 1000 Hz. The phase response for Ag/AgCl electrodes on wet skin was close to zero degrees between 100 Hz and 1000 Hz and was significantly lower (10 degrees) compared to the textile electrodes (40 degrees) between 1 Hz and 100 Hz. This is in complete agreement with the mechanistic principle of wet electrodes, where the resistive coupling is dominant. In contrast, the phase difference obtained for dry textile electrodes (40 degrees) suggests a combination of both resistive and capacitive coupling, indicative of the electrodes’ different transduction mechanisms. This analysis confirms the dominant resistive behavior for the Ag/AgCl electrodes compared to the dry textile electrodes, echoing the distinct mechanistic principles that guide the behavior of Ag/AgCl and dry electrodes [47].

The 3D knitted silver electrodes, designed with a specific geometry of 3 cm in diameter and 3 mm in height under a skin pressure condition of 30 mmHg, are selected for integration into the intelligent garment system. This choice is based on their proven lower impedance, which ensures enhanced signal quality and reliability in various environmental conditions. Their unique material composition and optimized design allow for superior performance in both dry and wet conditions, positioning them as the most appropriate for reliable, long-term biopotential monitoring within intelligent garments. The electrodes’ ability to adapt and perform consistently across different skin conditions underscores their optimal suitability for advanced wearable health technologies.

## 4. Comparative Analysis of 3D Knitted Silver Electrodes and Ag/AgCl Electrodes in ECG Monitoring

Following a series of impedance tests, our custom-designed 3D knitted silver electrodes have demonstrated exceptionally low impedance values. Notably, these values were significantly reduced under wet skin conditions compared to dry skin conditions. These promising results prompted us to further investigate the performance of the 3D knitted silver electrodes in capturing an electrocardiogram (ECG) signal. For this purpose, we utilized the hardware described in the previous sections for our intelligent garment system to conduct the monitoring.

**Figure 15:**
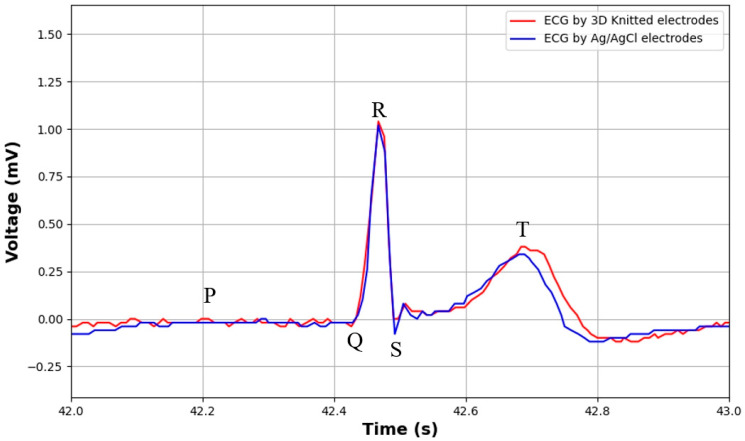
ECG signals recorded over 1 s using different electrodes: Ag/AgCl electrode (blue) and 3D knitted electrode (red).

The ECG data obtained from our experiments are illustrated in Figure 15. The figure shows the ECG signals recorded over a 1-s interval using both the 3D knitted silver electrodes (red) and the Ag/AgCl electrodes (blue). The electrodes were placed by having the subjects wear our intelligent garment system, and three gel electrodes were positioned in adjacent locations with the same single-lead configuration to ensure consistent signal acquisition conditions. Due to the limited size and precision of the portable ECG sensors we applied, which cannot match the traditional medical-grade ECG sensors, the P-wave in the recorded waveforms is not prominently visible. Both sets of data underwent identical filtering procedures, following the methodology from the referenced study: an improved integer coefficients IIR filter was employed. This included a high-pass filter with a cutoff frequency of 0.5 Hz to eliminate baseline drift and a low-pass filter with a cutoff frequency of 40 Hz to remove high-frequency noise. Additionally, a notch filter was used to suppress the 50 Hz power line interference. The high-pass filter is typically used to remove low-frequency baseline wander, often caused by breathing and other movements, while the low-pass filter is effective for removing high-frequency noise that can interfere with signal interpretation. The notch filter is crucial for eliminating power line interference at 50 or 60 Hz, a common source of noise in ECG signals [48].

As observed in Figure 15, the ECG waveforms collected by both electrode types are relatively clear. However, it is noticeable that the ECG data obtained using the 3D knitted silver electrodes exhibit a slightly higher level of noise compared to that from the Ag/AgCl electrodes. To quantitatively evaluate these differences, we calculated the signal-to-noise ratio (SNR) for both sets of signals. The SNR was defined as the ratio of the averaged power of the desired ECG to that of noise and it was used to evaluate the noise level. Since we are comparing the performance of two types of electrodes, the desired ECG is the one recorded by the Ag/AgCl electrodes, and the noise is the difference between the ECG recorded by the 3D knitted electrodes and the desired ECG.

The computed SNR for the ECG signal recorded with the 3D knitted silver electrodes is 6.95 dB. This indicates that the 3D knitted silver electrodes are capable of achieving good signal acquisition, despite the slight increase in noise compared to the Ag/AgCl electrodes. These findings underscore the potential of the 3D knitted silver electrodes for use in wearable health monitoring systems. Despite the slight increase in noise, the overall performance in terms of signal clarity and SNR is commendable. Thus, the 3D knitted silver electrodes exhibit considerable reliability and efficacy in ECG monitoring.

## 5. Biomeasurement Results

**Figure 16:**
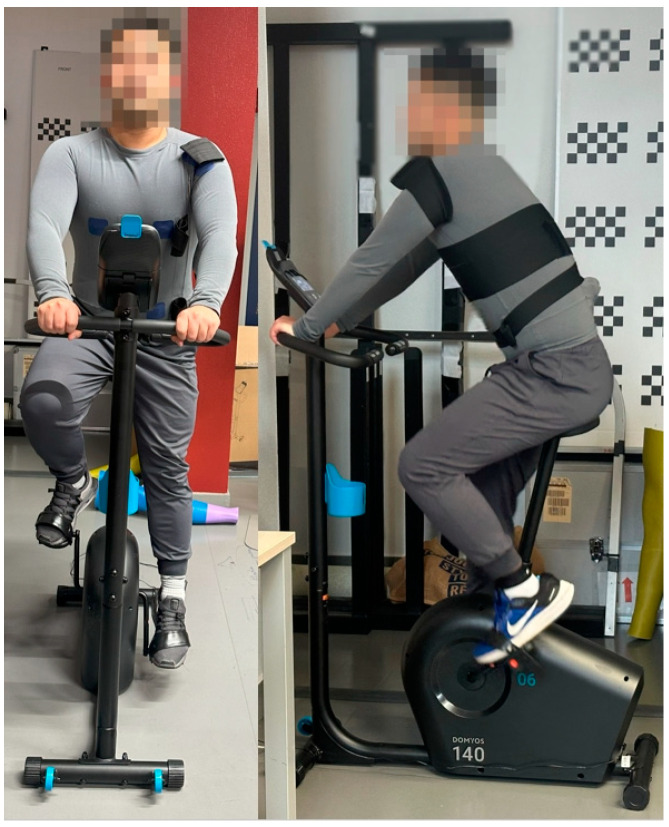
Cycling biomeasurement.

As shown in Figure 16, for the biomeasurement test, we employed the Essential exercise bike EB 140 (Decathlon, Villeneuve D’ascq, France) to conduct a cycling protocol designed to induce fatigue for optimal signal acquisition. Our method involved three cycling sequences, each comprising a dual-phase approach: a 7-min ride at a leisurely 5 km/h pace to establish a baseline physical state, immediately followed by a 3-min sprint at 30 km/h to rapidly induce fatigue. The total cycling time was 30 min. This transition from a moderate to high-intensity workload, without intervening rest periods, was aimed at mimicking real-world athletic conditions where varied exertion levels occur in succession, thereby creating an ideal setting for collecting ECG and EMG signals under conditions of heightened physical stress. The rationale behind this design is to challenge the electrode system’s ability to maintain signal integrity amidst perspiration, rapid movement, and fluctuating contact pressures, commonly experienced during intense physical activity. Furthermore, the uninterrupted nature of the test better reflects the continuous exertion athletes experience during training or competition.

The choice to forego rest periods between phases allowed for continuous data collection, reflecting the dynamic nature of physical exertion and its impact on biometric signal reliability. This approach, emphasizing immediate changes in intensity, is rooted in exercise physiology, offering a nuanced view of how varying exertion levels can enrich data analysis. By directly transitioning between slow and fast cycling speeds, our protocol aimed to simulate a realistic range of physiological responses, enhancing this study’s relevance to actual sports and exercise scenarios. This methodology ensures that our findings on the performance of 3D knitted silver electrodes in biometric monitoring are applicable to continuous, real-world athletic activities, closely mirroring the conditions athletes typically face.

**Figure 17:**
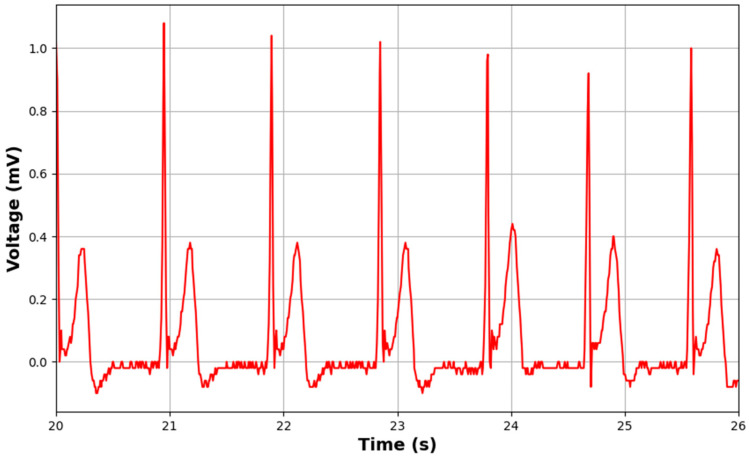
ECG signals obtained during cycling biomeasurement over a 6-s interval.

**Figure 18:**
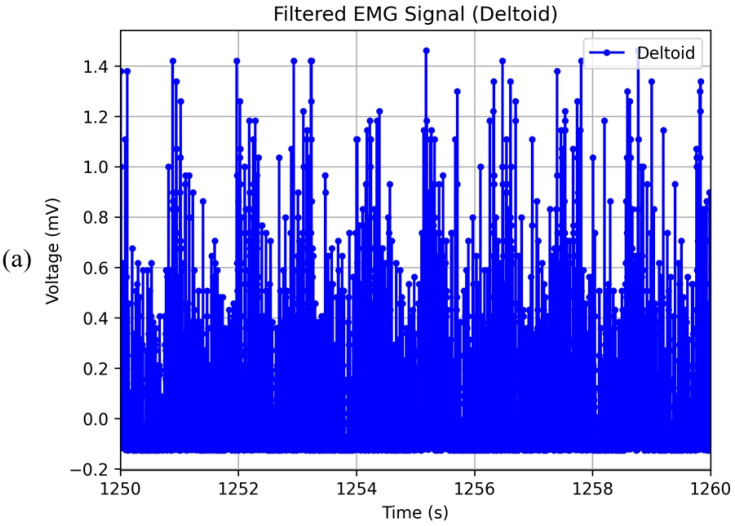

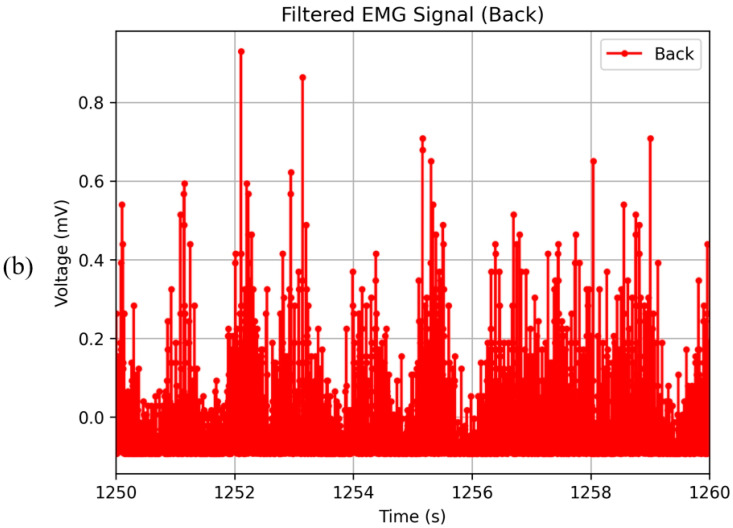
EMG signals obtained during cycling biomeasurement. (**a**) Deltoid EMG values for 10 s. (**b**) Back EMG values for 10 s.

Figure 17 and Figure 18 depict the ECG and EMG signals acquired during cycling biomeasurement, respectively. From Figure 16, it is evident that our intelligent garment system is capable of capturing clear ECG signals throughout the cycling process, thanks to the low impedance and tight skin integration of our specially designed 3D silver electrodes.

Figure 18 illustrates the EMG signals collected over a 10-s interval during cycling biomeasurement. Figure 18a shows the deltoid EMG values, while Figure 18b presents the back EMG values. The data shown have been processed through several filtering steps to ensure signal clarity and accuracy. Initially, we removed the DC offset by subtracting the mean value from the data. Subsequently, we applied a bandpass filter with an order of 2 and cutoff frequencies of 5 Hz and 125 Hz to filter out noise. This bandpass filtering step is crucial for eliminating low-frequency noise, such as movement artifacts, and high-frequency noise that could interfere with the signal interpretation.

After bandpass filtering, we performed full-wave rectification on the filtered signal to convert all negative values to positive, which helps in highlighting the overall amplitude of the EMG signal. Following rectification, we applied a low-pass filter with an order of 2 and a cutoff frequency of 3 Hz to smooth the signal and remove any remaining high-frequency noise, ensuring that only the relevant muscle activation patterns are retained. These waveforms clearly indicate changes in muscle electrical signals. Since the activity involves cycling, the muscles monitored are primarily in the upper limbs, which are not actively engaged in contraction, like in strength exercises. Consequently, the EMG patterns are less pronounced compared to those of muscles undergoing active contraction. However, the variations in muscle electrical signals are still discernible. Comparatively, the deltoid EMG values are significantly higher than the back EMG values. This disparity is attributed to the greater exertion of the anterior deltoid muscles compared to the erector spinae muscles during cycling. Another plausible reason is the greater compression applied by the intelligent garment system on the anterior deltoid region, leading to a counteractive response in the muscle, which is reflected by higher EMG values.

**Figure 19:**
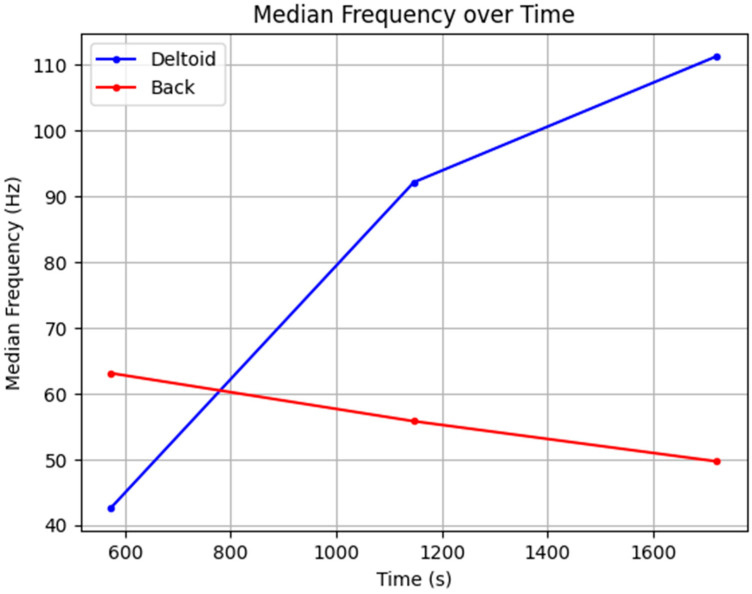
Median frequency of deltoid and back EMG.

To further assess muscle fatigue during this activity, we computed the median frequency (MF) for both muscle groups. The MF is a widely recognized indicator of muscle fatigue, representing the frequency at which the power spectrum is divided into two equal halves [49,50]. This metric provides insight into the shift in frequency components as muscles fatigue. Figure 19 illustrates the median frequency of the deltoid and back muscles throughout the cycling session. It is evident that only the median frequency of the erector spinae muscle decreases over time, while the median frequency of the anterior deltoid muscle remains stable. This observation suggests that the erector spinae muscles experience fatigue during cycling, whereas the anterior deltoid muscles do not.

These findings demonstrate the efficacy of our intelligent garment system, equipped with 3D silver electrodes, in capturing precise biometric signals and consistently monitoring muscle activity and fatigue. The variations in EMG values and the trends in median frequency align with physiological expectations and corroborate the hypothesis that different muscles respond differently to sustained activity.

Additionally, our system’s ability to provide continuous and reliable EMG monitoring during physical activity offers a valuable tool for further research into adaptive algorithms. These algorithms can potentially provide real-time feedback to athletes, guiding them to adjust their effort or technique to maintain optimal performance levels. While Ag/AgCl electrodes are standard for static measurements, their practicality during dynamic activities like cycling is limited due to issues such as electrode detachment caused by sweating and movement, and the risk of skin irritation with prolonged use. Our 3D silver electrodes provide a more reliable and skin-friendly alternative for continuous biometric monitoring during physical activity.

These comprehensive analyses of EMG signals highlight the potential of our intelligent garment system in wearable health monitoring, paving the way for advancements in real-time monitoring and feedback systems to enhance athletic performance and safety.

## 6. Discussion

The fabrication and integration of 3D biopotential electrodes into intelligent garments, as explored in this study, highlights a methodical approach to enhancing biopotential monitoring through advanced textile engineering. Utilizing STOLL’s M1 Plus 7.2 software and STOLL 72G knitting machine, we demonstrate the creation of complex 3D knitted structures, incorporating silver-plated nylon threads for electrodes and high-elasticity filaments for the garment’s other components. This blend of materials and knitting techniques not only ensures lower impedance for improved signal quality but also maintains the garment’s comfort and flexibility. The systematic impedance experiments, focusing on varying electrode diameters and heights, underline the critical importance of electrode geometry in achieving optimal signal detection. Additionally, the garment design, tailored for dynamic sports environments, strategically places electrodes to monitor vital signs without hindering performance, leveraging innovative designs for the seamless integration of technology and wearability. This approach signifies a significant step forward in developing wearable technologies that do not compromise on functionality or user comfort, setting a new standard for intelligent sports monitoring systems.

This paper’s findings from impedance measurements on 3D knitted silver electrodes reveal crucial insights into the optimization of electrode design for biopotential monitoring. It was observed that impedance decreases with frequency increase and rises with electrode height augmentation, highlighting the significant impact of electrode geometry on performance. This sensitivity underscores the need for precise electrode customization to enhance signal quality. Furthermore, the minimal impedance variation across certain electrode dimensions suggests an optimal range for design parameters, beyond which improvements may plateau. Comparatively, 3D knitted electrodes demonstrated superior performance under varying skin conditions against traditional Ag/AgCl electrodes, indicating their potential for reliable, long-term monitoring in both dry and wet conditions. These insights emphasize the importance of considering both physical and environmental factors in the development of wearable health technologies, guiding future advancements in the field. Incorporating the results from our impedance measurements, we determined the optimal parameters for electrodes suitable for the intelligent garment system to be a diameter of 3 cm and a height of 3 mm under a skin pressure condition of 30 mmHg. This selection is based on observed trends and comparisons, which indicated that these dimensions offer the best balance between impedance performance and user comfort under varying conditions, thereby enhancing the reliability of biopotential monitoring in real-time applications. This choice underscores the importance of tailored electrode design in developing advanced wearable health monitoring systems.

Following three sets of fatigue cycling biometric tests, our electrodes were able to accurately monitor ECG and EMG signals, with electromyographic signals indicating clear signs of fatigue. This confirms the effectiveness of our 3D knitted silver electrodes in capturing signals, particularly during dynamic activities and sweating conditions. The introduction of these 3D silver electrodes holds potential for broad market applications, extending beyond cycling to include a variety of long-term sports such as running and skiing. This capability for monitoring fatigue and performance could help in preventing injuries related to overexertion and contribute positively to scientific training methodologies. The findings suggest that our approach in designing wearable health technologies can offer reliable biopotential monitoring in real-time sports scenarios, emphasizing the importance of electrode design and material selection in achieving high-quality signal detection without compromising wearer comfort.

## 7. Limitations and Future Research Directions

The intelligent garment system presented in this study is designed specifically for sports monitoring, and it does not meet the stringent requirements necessary for medical applications, particularly in terms of high sampling frequency and denoising procedure due to the size of electronic components. As a result, the signals acquired by this system are insufficiently accurate for medical monitoring purposes and can only be used for fatigue prediction and the acquisition of basic physiological indicators at this stage. Additionally, our current hardware design has not yet achieved complete flexibility, especially regarding the sensors and power supply units, which leaves substantial room for improvement in terms of wearing comfort.

Future research in wearable physiological signal monitoring should focus on developing hardware by using a flexible, small-size device with higher sampling frequency and precision. Enhancements should also be made to improve resistance to data acquisition slippage and motion artifacts caused by intense physical activities, thereby enhancing both anti-interference capabilities and collection stability. Furthermore, advancements in real-time signal processing will require the development of more powerful processors capable of handling data collection independently, without the need for external computers. This would minimize signal transmission loss to the greatest extent possible, thereby improving overall system performance. Additionally, future directions include the application of AI technologies to achieve intelligent real-time fatigue prediction. We are currently working on this and expect to publish our findings in our next paper.

## 8. Conclusions

This study underscores the integration of 3D biopotential electrodes within an intelligent garment system, marking a significant advancement in long-term sports monitoring. The use of low-resistance silver fibers and sophisticated knitting techniques has led to the development of electrodes that significantly reduce skin-to-electrode impedance, enhancing the quality of signal acquisition.

The exploration of 3D knitted silver electrodes and their incorporation into an intelligent garment system represents a pivotal step forward in wearable technology for sports monitoring. By achieving low skin-to-electrode impedance and ensuring user comfort, this study provides a viable solution to the longstanding challenges of long-term physiological monitoring. It opens new avenues for real-time, continuous assessment of athletic performance and health, with potential applications extending beyond sports to include healthcare and daily life monitoring. Future work should focus on further refining electrode design and integration techniques to enhance the efficacy and accessibility of intelligent garment systems for a wider range of activities and user needs.

## Data Availability

The data presented in this study are available on request from the corresponding author.
